# Conquering today’s health paradox with the power of HEAL – an expert consensus report plus research priorities and policymaker roadmap

**DOI:** 10.3389/fpubh.2026.1695757

**Published:** 2026-03-20

**Authors:** Katharina C. Wirnitzer, Mohamad Motevalli, Derrick R. Tanous, Clemens Drenowatz, Maximilian Moser, Holger Cramer, Thomas Rosemann, Karl-Heinz Wagner, Andreas Michalsen, Beat Knechtle, Zlatko Fras, Adilson Marques, Nataša Fidler Mis, Fatima C. Stanford, Christian Schubert, Nandu Goswami, Claus Leitzmann, Per Morten Fredriksen, Gerhard Ruedl, Rodrigo A. Lima, Christian Kessler, Michael Jeitler, Naim A. Khan, Hassan Joulaei, Maryam Fatemi, Karl W. Kratky, Kara K. Palmer, Bernd Haditsch, Boštjan Jakše, Walter Kofler, Tomas Pfeiffer, Kathya L Cordova-Pozo, Patrizia Tortella, Simon Straub, Heidi Lynch, Manuel Schätzer, Anupama Krishnan, Shahnaz Fathima, Lukas Gatterer, Fabian Kriwan, Mittal Abhishek, Hemant Nandgaonkar, Shalaka Nandgaonkar, Abiola O. Adedara, Josep M. Haro, Amir Rashidlamir, Madan Thangavelu, Gonza Ngoumou, Éva Perpék, Michael Klaper, Bhaswati Bhattacharya, Werner Kirschner, Kathelijne M. H. H. Bessems, Peter Jones, Gregory Peoples, Raul Bescos, Christina Duftner, Georg Seifert

**Affiliations:** 1 Department of Pediatric Oncology and Hematology, Otto-Heubner Centre for Paediatric and Adolescent Medicine (OHC), Charité – Universitätsmedizin Berlin, Berlin, Germany; 2 Charité Competence Center for Traditional and Integrative Medicine (CCCTIM), Charité – Universitätsmedizin Berlin, Berlin, Germany; 3 Department of Secondary Education, University College of Teacher Education Tyrol, Innsbruck, Austria; 4 Department of Sport Science, Leopold-Franzens University of Innsbruck, Innsbruck, Austria; 5 Division of Sport, Physical Activity and Health, University of Education Upper Austria, Linz, Austria; 6 Division of Physiology & Pathophysiology, Otto Loewi Research Center for Vascular Biology, Immunology, and Inflammation, Medical University Graz, Graz, Austria; 7 Human Research Institute, Weiz, Austria; 8 Institute for General Practice and Interprofessional Care, University Hospital Tübingen, Tübingen, Germany; 9 Robert Bosch Center for Integrative Medicine and Health, Bosch Health Campus, Stuttgart, Germany; 10 Institute of Primary Care, University of Zurich, Zürich, Switzerland; 11 Department of Nutritional Sciences, Faculty of Life Sciences, University of Vienna, Vienna, Austria; 12 Research Platform “Active Ageing,” University of Vienna, Vienna, Austria; 13 Institute of Social Medicine, Epidemiology and Health Economics, Charité - Universitätsmedizin Berlin, Corporate Member of Freie Universität Berlin and Humboldt-Universität zu Berlin, Berlin, Germany; 14 Department of Internal Medicine and Nature-Based Therapies, Immanuel Hospital Berlin, Berlin, Germany; 15 Medbase St. Gallen, St. Gallen, Switzerland; 16 Division of Medicine, University Medical Centre Ljubljana, Ljubljana, Slovenia; 17 Faculty of Medical, University of Ljubljana, Ljubljana, Slovenia; 18 CIPER, Faculdade de Motricidade Humana, Universidade de Lisboa, Lisbon, Portugal; 19 Núcleo de Investigación en Ciencias del Movimiento, Universidad Arturo Prat, Iquique, Chile; 20 Independent Researcher, Ljubljana, Slovenia; 21 Massachusetts General Hospital, MGH Weight Center, Department of Medicine-Division of Endocrinology-Neuroendocrine, Department of Pediatrics-Division of Endocrinology, Nutrition Obesity Research Center at Harvard (NORCH), Harvard Medical School, Boston, MA, United States; 22 Department of Psychiatry, Psychotherapy, Psychosomatics and Medical Psychology, Medical University of Innsbruck, Innsbruck, Austria; 23 Center for Space and Aviation Health, Mohammed Bin Rashid University of Medicine and Health Sciences, Dubai, United Arab Emirates; 24 Institute of Nutrition, Justus Liebig University Giessen, Giessen, Germany; 25 Faculty of Social and Health Sciences, University of Inland Norway, Elverum, Norway; 26 Faculty of Health, Welfare, and Organization, Østfold University College, Halden, Norway; 27 Parc Sanitari Sant Joan de Déu, IRSJD, CIBERSAM, Sant Boi de Llobregat, Barcelona, Spain; 28 Department of Integrative Health Care and Prevention, University of Augsburg, and University Hospital Augsburg, Augsburg, Germany; 29 Faculté des Sciences de la Vie, Physiologie de la Nutrition & Toxicologie (NUTox), UMR 1231 INSERM/UB/AgroSup, Université de Bourgogne, Dijon, France; 30 Health Policy Research Center, Institute of Health, Shiraz University of Medical Sciences, Shiraz, Iran; 31 HIV/AIDS Research Center, Voluntary Counseling and Testing Center, Institute of Health, Shiraz University of Medical Sciences, Shiraz, Fars, Iran; 32 Faculty of Physics, University of Vienna, Vienna, Austria; 33 School of Kinesiology, University of Michigan, Ann Arbor, MI, United States; 34 Preventive Medical Examination and Screening Centre, Austrian Health Insurance Fund ÖGK – Österreichische Gesundheitskasse, Graz, Austria; 35 Independent Consultant, Kranjska Gora, Slovenia; 36 Department Normal Physiology, I.M. Sechenov Moscow State Medical University (Sechenov University), Moscow, Russia; 37 Institute for Hygiene and Medical Microbiology, Medical University of Innsbruck, Innsbruck, Austria; 38 Institute for TCIM/CAM, Science and Research Department, Prague, Czechia; 39 Professional Chamber Sanator – the Union of Biotronicists of Josef Zezulka, Prague, Czechia; 40 Department of Research Methodology, Institute for Management Research, Radboud University, Nijmegen, Netherlands; 41 Department of Human and Social Sciences, University of Enna “Kore”, Enna, Italy; 42 Department für Kinder- und Jugendheilkunde, Pädiatrie I, Gastroenterologie und Hepatologie, Universitätskliniken Innsbruck, Tirol Kliniken, Innsbruck, Austria; 43 Department of Kinesiology and Health Sciences, Point Loma Nazarene University, San Diego, CA, United States; 44 Special Institute for Preventive Cardiology and Nutrition (SIPCAN), Salzburg, Austria; 45 Department of Swasthavritta (Ayurvedic Branch of Preventive Medicine and Community Health), VPSV Ayurveda College Kottakkal, Kerala University of Health Sciences, Thrissur, Kerala, India; 46 Department of Orthopaedics and Trauma Surgery, University Hospital of St. Pölten, St. Pölten, Austria; 47 Department of Anaesthesia and Intensive Care, University Hospital of Innsbruck/Tirol Kliniken GmbH, Medical University Innsbruck, Innsbruck, Austria; 48 Amity Institute of Public Health, Amity University, Noida, Uttar Pradesh, India; 49 Department of Surgery, Vardhman Mahavir Medical College and Safdarjung Hospital, New Delhi, India; 50 Occupational Therapy, Seth GS Medical College & KEM Hospital, Maharashtra University of Health Sciences (MUHS), Mumbai, India; 51 Occupational Therapy, Hands on Therapy Concepts Pvt. Ltd., Mumbai, India; 52 Department of Prevention, Care and Treatment (PCT), Institute of Human Virology Nigeria, Abuja, Nigeria; 53 Department of Exercise Physiology, Faculty of Sport Sciences, Ferdowsi University of Mashhad (FUM), Mashhad, Iran; 54 Ayush Valley Foundation, Shoranur, Kerala, India; 55 Mind-Matter Unification Project, Theory of Condensed Matter Group, Cavendish Laboratory, Department of Physics, University of Cambridge, Cambridge, United Kingdom; 56 ELTE Centre for Social Sciences, Hungarian Academy of Sciences Centre of Excellence, Budapest, Hungary; 57 Moving Medicine Forward, Medical School Nutrition Education Initiative, St. Peterburg, FL, United States; 58 Department of Medicine, Weill Cornell Medical College, New York, NY, United States; 59 Center for Ayurveda Studies, Indic Academy, Hyderabad, Telangana, India; 60 Department of Health Promotion, Faculty of Health, Medicine and Life Sciences, NUTRIM Institute of Nutrition and Translational Research in Metabolism, Maastricht University, Maastricht, Netherlands; 61 NHS Professionals, University of Greater Manchester, Wigan, United Kingdom; 62 Faculty of Science, Medicine, and Health, Centre for Medical and Exercise Physiology/Graduate Medicine/School of Medicine, University of Wollongong, NSW, Australia; 63 Faculty of Health, School of Health Professions, University of Plymouth, Plymouth, United Kingdom; 64 Clinical Division of Internal Medicine II, Department for Internal Medicine, Medical University of Innsbruck, Innsbruck, Austria; 65 Departamento de Pediatria, Faculdade de Medicina, Instituto de Tratamento do Câncer Infantil (ITACI), Universidade de São Paulo, São Paulo, Brazil

**Keywords:** non-communicable disease, physical activity, physical exercise, plant-based, policy, prevention, public health, vegan

## Abstract

**Background:**

Despite growing scientific evidence and health guidelines, the global health paradox persists, with rising lifestyle-related diseases and escalating healthcare costs exposing the inadequacy of current efforts.

**Objective:**

Three multidisciplinary congresses were held to generate evidence-based conclusions aimed at addressing the global health paradox.

**Methods:**

A total of 58 experts from 62 entities participated in the international research and knowledge-exchange panels. Experts reviewed the latest findings to develop practical strategies and identify key research and policy priorities, focusing on the Healthy Eating & Active Living (HEAL) approach.

**Results, conclusion, and relevance:**

The expert consortium endorsed a 33 evidence-based consensual-statement policy roadmap for addressing global health challenges, emphasizing that the HEAL approach can significantly contribute to the “Prevention First” appeal and broad ethical, social, ecological, and economic advantages, and ultimately supporting policy change.

## Highlights

- *Question:* How can evidence-based strategies effectively improve individual health outcomes and thus reduce the prevalence of NCDs and the public health burden?
- *Approach:* This consensus-statement report provides conclusions from three international events that addressed today’s global health paradox on why growing advances in medicine and increasing healthcare budgets cannot control the ever-rising prevalence of NCDs.
- *Output:* The panel of 58 experts discussed neglected public-health areas and concluded with 33 evidence-based consensus statements along with a policy roadmap.
- *Impact:* Lifestyle medicine holds significant potential to contribute to the urgent need for a pan-governmental and pan-organizational “Prevention First” approach to treat and cure with joint efforts for synergistic effects.
- *Science-to-Policy enables Health in All Policies (HiAP):* It is the consensus of the expert panel that the power of personal lifestyles can significantly contribute to the “Prevention over Therapy” appeal, with the dual HEAL (Healthy Eating & Active Living) approach to sustainable and lifelong health and care being the minimum recommendation to unlock the power of lifestyles (behaviors and habits) to promote better individual health and thereby shape stronger public health for nations (1).

## 1 Introduction

Over the past decades, the world has witnessed a shift in the primary focus of health concerns from infectious to chronic diseases, specifically non-communicable diseases (NCDs) (2, 3). Currently, NCDs account for 74% of all deaths globally (4). To control health problems, governments and international health organizations have established various policies that have led to a general increase in healthcare budgets worldwide, and are projected to cumulate to USD 15–44 trillion by 2050 (5–7). In addition, there has been rapid growth in the quantity and quality of health and medical investigations over the past decades (8). Despite groundbreaking accomplishments in science and technology over the past decades, such as sanitization and hygiene, vaccination programs, and cancer screening (9–11), there are still gaps between the practical understanding of disease causes, the identification of biological markers of their presence and stage, and specific indicators influencing the effectiveness of potential solutions (12, 13). While the theoretical aspects of modern medicine imply the necessity of applying the three principles (diagnosis, therapy, and prognosis) to tackle the burden of disease (14), many current health approaches, specifically those to manage NCDs, are based on clear-cut diagnoses that often miss the underlying causes and subtler manifestations of illness (13–15).

The impact of different health-determining domains is not directly associated with the amount of money spent annually. In particular, individual behavior plays the most significant role in human health (contributing 38%), whereas the US spends 13 times more on medical care, which constitutes only 11% of overall health outcomes (16). Data show that prescribed medications rank as the third leading cause of death, following heart disease and cancer, in Western countries, with approximately half of these deaths due to taking the medication correctly as prescribed, while the other half of these deaths are due to overdose or drug abuse (17, 18). Therefore, it seems crucial to step back from a fragmented and merely reactive health approach, which involves minimal interactions among specialists, often resulting in non-concerted prescriptions and scattered follow-ups.

The desire for a happy and long life has been a fundamental human aspiration and traces back to the beginning of humankind (19). In 1550, however, Luigi Cornaro (an Italian humanist, 1,475–1,566) reported that the human lifespan could be extended by making lifestyle modifications (20). This idea is consistent with the current body of science, which indicates that genetics accounts for 20–25% of an individual’s lifespan, while various factors determine the remaining 75–80%, with lifestyle being the largest contributor among them (21, 22). Currently, however, lifestyle medicine is a well-defined, evidence-based, and comprehensive approach to preventing, treating, and even reversing various diseases through the implementation of health-promoting behaviors and the replacement of poor and unhealthy ones. Lifestyle medicine taps the potential for healthy dietary habits, regular physical activity (PA), stress-management techniques, positive relationship reinforcement (solid social support), improved sleep quality, and restricted smoking and alcohol intake (23, 24). By strongly emphasizing health-related behavior, lifestyle medicine may reduce dependence on medical treatments such as pharmaceutical therapy (prescribed medication) or surgical strategies, as well as the potential side effects that may result from interventions and treatments (23, 24).

Health professionals and healthcare systems face various challenges (e.g., financial interests, dissemination of conclusive research) in tackling public-health issues, which complicate critical efforts to control and reduce the prevalence of NCDs and their underlying risk factors (1–4). Therefore, this consensus report aimed to establish direct statements along with a policy roadmap to address today’s global health paradox of why growing advances in health science and increasing healthcare budgets are not sufficient to stop the increasing prevalence of NCDs.

## 2 Methods

By bringing together experts and health professionals from diverse areas through common ground, the current health issues of pressing concern could be discussed. The present paper reports the critical consensus of theme-specific results from three international, multidisciplinary, and cross-university events (25, 26), which hosted 284 internationally recognized experts from 76 universities, stakeholders, and organizations (including the WHO Regional Office for Europe) across 31 nations and 5 continents:

(1) “Health and Wellbeing: Addressing Today’s Global Paradox. Visioning an International Research & Knowledge Exchange.” 6 February 2020, one-day hybrid conference, Innsbruck, Austria.
(2) “Improving Child & Adolescent Health for Better Public Health – Fiction or Within the Scope of Possibility? The perspective of a lifestyle-centered approach for Addressing Today’s Global Health Paradox.” 10–11 November 2020, two-day e-conference, Innsbruck, Austria.
(3) “The future in (y)our hands. Improved public health arises through the better health of every individual. A tertiary education symposium about the future of human and planetary health.” 8 June 2022, one-day hybrid conference, Innsbruck, Austria.

**Figure 1:**
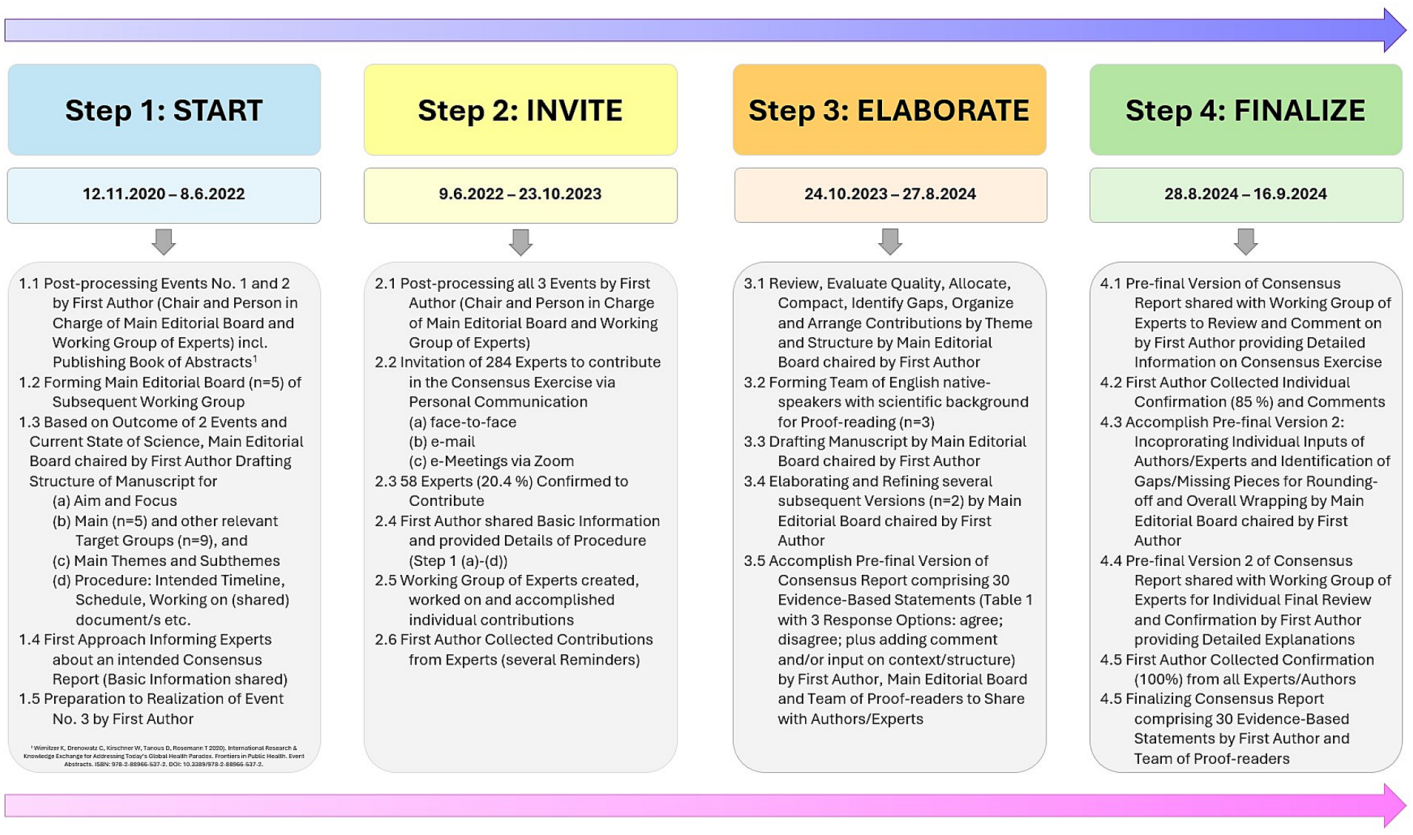
Flowchart of the consensus exercise process based on the ACCORD-method “Consensus Meeting” for health-related activities and research (27). © Katharina C. Wirnitzer.

Through a consensus-generating approach using the ACCORD-method “Consensus Meeting” for health-related activities and research (27) (Figure 1; Supplementary Appendix 1), chaired by the first author in charge (KCW), 58 out of 284 experts (20.42%) from 62 universities, organizations, and stakeholders across 20 nations and 5 continents formed the working group. The working group’s activities included in-person meetings of the main editorial board of physicians, public health experts, and sports scientists (KCW, MM, DRT, CD, and WKo), with senior experts in their respective fields were engaged through structured remote exchanges throughout the entire consensus process. They contributed to the consensus exercise process (12 November 2020–16 September 2024): (i) discussed the latest scientific findings, including existing limitations in health approaches, (ii) identified gaps and untapped potentials in encouraging basic approaches that have been grossly neglected, (iii) debated the necessity of specific research priorities, (iv) foresighted policies supporting novel and promising scientific and practical efforts, and (v) weighed in developing practical strategies to promote healthy lifestyle behaviors in everyday scenarios at different levels (individual, community, and policy-oriented) and settings with a specific emphasis on holistic and integrated health perspectives, particularly the preventative dual approach of Healthy Eating & Active Living (HEAL) (28–31) for sustainable, lifelong health and wellbeing.

## 3 Results

The 33 evidence-based statements developed by this consortium of experts encompass various topics concerning sustainable health behavior and improving global health, including general considerations, health education and literacy, guidelines, and priorities for future research (Table 1), along with a policy proposal that includes a roadmap to address today’s health paradox (Table 2). These statements provide an in-depth overview of the essential lifestyle-oriented strategies involved in promoting and maintaining good health through behavior, focusing on the dual approach of sustainable, lifelong health and wellbeing, including the associated educational and communal considerations of the simple formula of HEAL.

**Table 1:** Summary of the 33 consensus statements/recommendations.

Statements		Target action group	Level of evidence*
Part 1. General aspects of health behavior (*n* = 9)			
1	The definition of health is fundamentally rooted in holistic wellbeing, which emphasizes the importance of directing both preventive and promotive health approaches.	Researchers, clinicians, policymakers, journalists, and educational institutions	5
2	Addressing the detrimental effects of chronic unhealthy behaviors throughout one’s lifespan requires the adoption of healthy lifestyle behaviors, and merely relying on short-term therapeutic interventions, such as pharmacotherapy or surgical techniques, is neither adequate nor permissible.	Clinicians, policymakers, public health, and educational institutions	5
3	In addition to the six pillars of lifestyle medicine, various other health behaviors can help improve physical, mental, and emotional health: managing screen time, engaging in PA and exercise, spending time in nature, practicing gratitude, practicing artistic activities, and maintaining good hygiene.	Researchers, clinicians, policymakers, and educational institutions	4
4	The resilient nature of some lifestyle-related issues and problems, such as those associated with chronic stress, may not exhibit acute signs but can have negative impacts on a specific future state of health (e.g., in psychosomatic and/or psycho-neuro-immunological conditions).	Researchers, clinicians, and educational institutions	5
5	Initiating evidence-based policies and advocating for direct and indirect legislation that promotes innovative, holistic, and lifestyle-oriented health behaviors, while remaining aligned with evidence-based clinical care, is essential for facilitating sustainable implementation in everyday life.	Policymakers, journalists, public health, health-related industries, and educational institutions	5
6	Regular seamless assessment of health status, behaviors, and habits, including body composition, physiological parameters (such as clinical biomarkers and underlying mechanisms), and mental and psychological wellbeing, indirectly motivates target populations toward enhancing their commitment to health promotion plans and measures.	Clinicians, practitioners, policymakers, community organizations, public health, and educational institutions	5
7	Private and public sector companies and communities should prioritize promoting healthy lifestyles among their members, including encouraging participation in worksite health and wellbeing programs. Such initiatives can benefit individual and public health and employee efficiency at work, resulting in a return on fiscal health investments in human resources for the business and the public sector.	Policymakers, community organizations, public health, and health-related industries	5
8	Enhancing access to healthy lifestyles, environments, and opportunities and programs in diverse educational and community settings and contexts is essential for promoting healthy habits and behaviors among target populations. Practical decisions considering hands-on solutions should be made to achieve this goal.	Policymakers, community organizations, public health, and educational institutions	5
9	As a feasible, cost-effective approach for individuals of all ages, HEAL is a practical and sustainable method to attain good health. This dual approach, as a minimum recommendation for lifelong health, is most promising because it is not only beneficial for individual health but also has the potential to shape and improve public health at the national level for future generations and is also advantageous for ethical, social, ecological, and economic reasons.	Clinicians, practitioners, policymakers, community organizations, public health, and educational institutions	1
Part 2. Healthy Eating & Active Living (HEAL) (*n* = 9)			
10	Healthy eating should be characterized by both the quantity and quality of one´s dietary pattern, which is generally translatable into two fundamental principles: (i) quantity: ensuring that energy intake does not usually exceed energy requirements; and (ii) quality: increasing the consumption of healthy fresh and unrefined foods, such as vegetables, fruits, beans, legumes and pulses, grains, tubers, nuts, seeds, and herbs, while also maintaining proper hydration (preferably by water).	Clinicians, practitioners, policymakers, community organizations, public health, and educational institutions	5
11	Organic, plant-based, whole-food items may provide significant health benefits, such as increasing the quality of diet by using healthy preparation methods (e.g., raw-food techniques, steaming, baking, and fermenting) to ensure a better supply of certain nutrients, preservation of nutrients, and promotion of the bioavailability of phytochemicals while avoiding (ultra-)processed and preserved foods and reducing exposure to harmful substances such as synthetic pesticides in parallel.	Clinicians, practitioners, community organizations, policymakers, public health, and educational institutions	1
12	To meet active living standards, it is recommended that children be physically active for at least 60 min/day. Adults should engage in 150–300 min/week of moderate-intensity PA and exercise (or 75–150 min/week of vigorous-intensity aerobic PA), including muscle-strengthening activities on at least 2 days/week.	Clinicians, practitioners, policymakers, community organizations, public health, and educational institutions	1
13	To establish a regular PA routine and maximize health-related benefits, guidelines for weekly PA can be translated into daily 20–30 min moderate-to-high-intensity workouts while incorporating “bonus” PA into daily routines of active mobility, such as taking the stairs instead of elevators, cycling or walking instead of driving, performing household chores, and gardening.	Clinicians, practitioners, policymakers, community organizations, public health, and educational institutions	5
14	Engaging in some PA is always preferable to being inactive. Individuals who struggle to meet the minimum level of PA recommendations should consider starting with less intense activities and gradually increasing the frequency, intensity, and duration of training sessions over time.	Clinicians, practitioners, community organizations, and educational institutions	5
15	Misconceptions exist regarding participation in sports and exercise and adverse effects on specific health conditions, including asthma, hypertension, and joint problems. However, these beliefs lack scientific support and often arise from limited understanding regarding choosing the appropriate type, intensity, duration, and frequency of PA, sports and exercise, as well as the necessary precautions to take during exercise.	Clinicians, practitioners, policymakers, community organizations, public health, and educational institutions	5
16	Access to healthy food and regular PA is influenced by environmental, community-based, and societal factors, with safety concerns regarding food, water, air quality, and living conditions posing major barriers, particularly in under-resourced or densely urbanized areas.	Policymakers, community organizations, public health, and educational institutions	1
17	Affordability plays a crucial role in shaping dietary choices and PA, as healthy, fresh, and organic foods are often more expensive or less accessible in low-income communities, whereas limited access to safe and affordable exercise facilities further hinders adherence to health recommendations.	Policymakers, community organizations, and public health	1
18	Cultural preferences, traditions, and beliefs strongly influence food choices and PA, and public health strategies should be culturally sensitive and inclusive, promoting practices that respect local diets, cooking methods, and culturally meaningful forms of movement.	Clinicians, practitioners, policymakers, community organizations, public health, and educational institutions	1
Part 3. Health education and literacy (*n* = 5)			
19	The acquisition of thorough knowledge regarding healthy lifestyle behaviors and their integration into the personal lives of teachers, educators, trainers, lecturers, and academic staff is necessary. This enables them to effectively convey behavior to pupils and students and empowers them to serve as authentic, genuine, and exemplary role models.	Policymakers, community organizations, and educational institutions	5
20	In line with the advancement of science, it is crucial to frequently update the knowledge base and training programs related to healthy lifestyle behaviors for educators and healthcare providers in all educational settings, ranging from elementary to tertiary levels, especially in medical education.	Researchers, policymakers, and educational institutions	5
21	The development of modules, courses, and training on preventive and promotive health strategies in medical and nursing curricula represents a vital endeavor toward equipping future doctors, therapists, and other healthcare professionals with comprehensive, evidence-based knowledge and practical understanding of the importance of preventive interventions.	Clinicians, practitioners, policymakers, and educational institutions	5
22	School-based health education should extend beyond the classroom; practical environments such as school gardens, kitchens, labs, and public catering, including cafeterias, can also provide valuable and additional learning opportunities. To enhance efficacy, students should be given a degree of responsibility, allowing them to take on roles as activity leaders and supervisors in the design, preparation, and execution of programs.	Policymakers, community organizations, and educational institutions	1
23	School principals and educators should consider the pupil’s starting point and measure progress individually. To this end, children and adolescents should be engaged in health and movement activities that are appropriate for their current level of ability and skills, with a gradual increase in intensity as they advance toward higher levels of competencies and mastery.	Policymakers, community organizations, and educational institutions	5
Part 4. Research priorities and considerations in health behavior (*n* = 10)			
24	Interdisciplinary and multi-sectoral collaboration must be actively pursued in future research endeavors to develop effective, personalized, and comprehensive interventions, programs, and measures to promote healthful and sustainable lifestyle behaviors and habits.	Community organizations, public health, and educational institutions	5
25	Future research should prioritize customized strategies to promote the health and wellbeing of vulnerable populations, including infants, children, adolescents, young adults, and individuals with a history of NCDs, by developing effective interventions, guidelines, and policies that support healthy behaviors.	Researchers, journalists, policymakers, and educational institutions	5
26	Further research is necessary to investigate the potential indirect changes in healthy lifestyle behavior arising subsequently to other health behavior interventions. More differentiated study designs (standardized cut-off points) should be applied to distinguish the specific associations between a particular health parameter calculated at different levels (e.g., poor vs. acceptable vs. healthy sleep) and other health factors (e.g., diet, PA, stress, and substance use).	Researchers, and journalists	5
27	To accurately assess health behavior indicators, a comprehensive approach is required that incorporates self-reported data, objective measures, and biometric assessments to holistically understand the individual’s health behavior.	Researchers, and journalists	5
28	Future investigations should consider lower-prevalence lifestyle-related populations (such as vegans and vegetarians). Proper and up-to-date classification of these populations based on international references can lead to a better understanding of the health landscape and result in more effective healthy lifestyle approaches.	Researchers, and journalists	5
29	It is imperative for future studies to develop multi-component and multilevel study designs that enable the measurement of confounding effects rather than solely adjusting or controlling them in data analysis. To accurately assess health behavior indicators, it is also important to conduct more reliable assessments spanning multiple levels, from general health habits to corresponding biomarkers.	Researchers, and journalists	5
30	As diet and PA both constitute interwoven, related components of the energy balance equation, it is essential for research efforts to consistently investigate these two domains in a cohesive and integrated manner.	Researchers, and journalists	5
31	As technology dominates educational practices, there is an urgent need to frequently update and enhance relevant research data and guidelines, particularly concerning technology-related health behaviors, such as the educational and non-educational aspects of screen time use among children and adolescents.	Researchers, policymakers, health-related industries, and educational institutions	5
32	Additional research is necessary to investigate the monetary burden and financial consequences of unhealthy lifestyles and the economic advantages of health promotion programs. This requires differentiated assessments for each of the six lifestyle areas, as well as for the holistic lifestyle-medicine approach.	Researchers, policymakers, and journalists	5
33	As technology rapidly evolves, future scientists and policymakers should explore the potential benefits and limitations of technology in personalized medicine to enhance personalized healthcare delivery, including prioritizing the development and integration of cutting-edge technologies such as artificial intelligence and machine learning, as well as e-health tools, devices, and services.	Researchers, policymakers, journalists, public health, health-related industries, and educational institutions	5

Public health, public health experts/professionals. PA, physical activity. Strength of evidence: *Quality Rating Scheme of Level of Evidence for Studies and Other Evidence, modified from Oxford Center for Evidence-Based Medicine, with ratings from 1 (properly powered and conducted randomized clinical trial; systematic review with meta-analysis) to 5 (opinion of respected authorities; case reports).

**Table 2:** Policy roadmap (including 10 milestones) for policy and decision makers to unfold the power of lifestyle arising from HEAL – Healthy Eating & Active Living and to enable within HiAP – Health in All Policies (74).

No.	What →	← How →	← Who
1	State Mandate for the HiAP framework for nationwide health promotion and prevention of NCDs	a. Policy brief by law, decree, regulations, guidelines, etc. as foundation to enable and implement accorded cross-sectoral joint-forces and synergistic structures and actions. b. Coordinate across medical, educational, and policy systems.	National governments, relevant ministries and agencies, education, and related departments; international and intergovernmental bodies, entities, and organizations
2	Establish measures to regulate harmful marketing practices along with comprehensive monitoring and evaluation	a. Enforce guidelines on food and lifestyle product marketing, especially towards youth. b. Track outcomes and related output of measures/interventions to evaluate progress and guide future directions based on gaps and weaknesses. c. Adapt data-based strategies and sequence with latest scientific evidence.	Regulatory bodies and consumer protection agencies; Health Ministries (EU, international); Medical and Public Health policymakers; Public Health evaluators, health system administrators, and statutory health insurance entities
3	Upgrade and adapt curricular state mandates and tertiary education/training as (Child) Public Health tools	a. School curricula: primary and secondary levels, transformatory development from the compulsory subject “Physical Education” to “Lifestyle Education” leading school health promotion. b. University/college curricula: Nationwide sequencing of basic-to-advanced modules on HEAL for medical and pedagogical studies.	Department of Education; State and local education officials; Federal Ministries of Health, Education, Science, and Research; Universities, colleges, and community colleges
4	Advanced education and training and modules for health professionals	a. Embed lifestyle-related health content into education and training curricula for essential and continuous professional development. b. Cooperation between educational and medical institutions to successfully combine synergistic effects of relevant specialist disciplines and expertise.	Universities/medical schools; Public Health educators; advanced training institutions of medicine, therapy, sports/exercise, nutrition, life sciences, etc.
5	Transfer of evidence-based knowledge	Translate science-driven data to health professionals, mass media campaigns, and the public, providing the latest findings/information for individuals/patients, doctors/therapists, nutritionists, exercise scientists, and others.	National/Federal Ministries of Science and Research, Health, and Education; Nationwide occupational organizations
6	Enhance public caterers and school/university meals	Update nutrition guidelines and facilitate implementation in public institutions across all levels, such as schools, universities, hospitals, prisons, and others.	Ministries of Education, nutrition policymakers, governmental entities/bodies, and/or organizations
7	Improve infrastructure for promoting HEAL: school and public catering, PA and exercise community offers	Reassess offerings and transform infrastructural facilities providing access to unprocessed (e.g., organic) food and affordable cafeterias, and apply meal guidelines to improve community and local meal standards, active transport, and engaging offers for PA and exercise in safe and healthy public spaces.	Urban planners, local governments, and social workers; Management boards of public entities and bodies, e.g., kindergartens, schools, universities, hospitals, nursing facilities, senior residences, prisons, and others.
8	Support community-based, holistic health initiatives	Fund and promote regional and local lifestyle health programs tailored to community requirements and needs.	Community organizations, local NGOs, and municipal governments
9	Implement culturally adapted programs	Design community-level tailored HEAL measures and interventions that reflect cultural contexts and align with national health promotion strategies.	Local health departments, cultural associations, and community leaders
10	Clinical routines and standard medical check-ups integrate regular lifestyle assessment	Use validated tools for routine monitoring and evaluation, alongside personalized (i.e., age, gender) and individualized (person/patient) counseling.	Hospital administration, medical schools; primary/family care physicians and other health professionals, e.g., nutritionists, exercise scientists, statutory health insurances, healthcare providers

PA, physical activity.

Overall, the consensus outcomes span four domains (Table 1): Part 1 addresses general aspects of health behavior and foundational determinants of sustainable health and comprises nine statements; Part 2, HEAL, outlines integrated lifestyle-focused preventive strategies across nine statements; Part 3, health education and literacy, highlights the importance of structured, lifelong educational efforts and includes five statements; and Part 4, research priorities and methodological considerations, identifies pressing gaps and future directions spanning 10 statements. Together, these consolidated expert perspectives outline a coherent and actionable framework for strengthening population health across the lifespan.

## 4 Discussion

The benefits of healthy lifestyle behaviors are not limited to the promotion of individual health or the public health of nations. Each component of a healthy lifestyle considers ethical, social, and ecological aspects directly and indirectly (32–34). The United Nations’ Sustainable Development Goals (UN SDGs) mainly aim to end (#1) poverty and (#2) hunger globally (35). To do so, however, the most critical steps are achieving (#3) good health and wellbeing based on (#4) quality education (35), especially for women (36). From a broader perspective, personal health behavior is connected to planetary health and can significantly contribute to preserving and maintaining human development (34, 35). In parallel, ecosystem changes, particularly climate change (primarily originating from human behavior over the past century), pose a significant threat to human health (37–39). In addition to increasing the likelihood of natural disasters (39), anthropogenic climate change has gradually led to environmental alterations, such as air pollution, loss of biodiversity, resource depletion, more frequent and elevated heatwaves, deforestation, and exacerbated water shortages, which may result in a wide range of health concerns, including instability and insecurity of resources, particularly food insecurity (34, 36, 37). However, the implementation of holistic solutions that consider synergies and trade-offs to target the UN SDGs and simultaneously address health and climate change challenges is restricted by the lack of effective action by policy and decision-makers at both the national and global levels (38–41).

**Figure 2:**
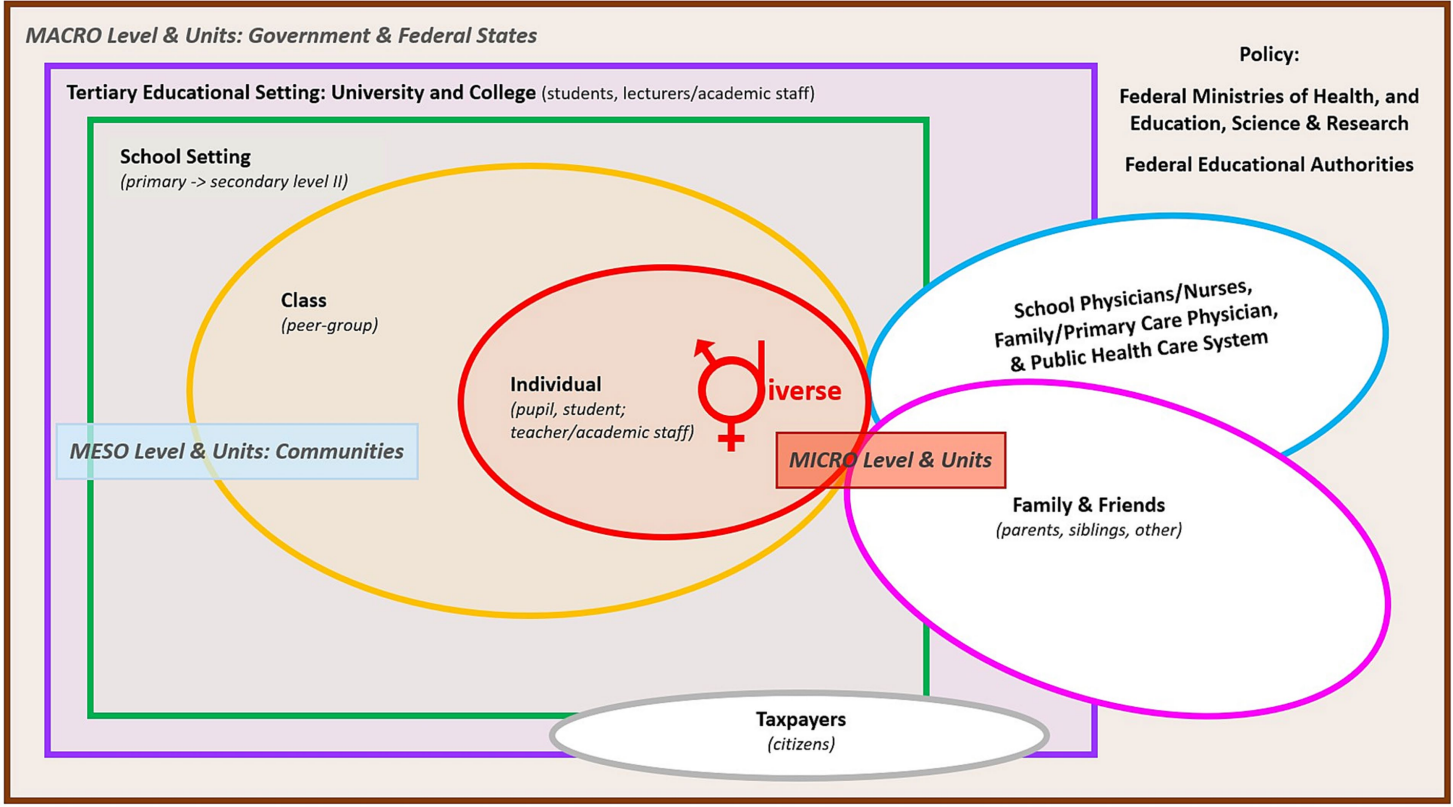
Target groups considering personal and public health to be addressed, from the micro-level of individuals and families over the meso-level of communities (e.g., schools, universities, regions) to the macro-level determined by state and federal policies, in accordance with “The Main Health Determinants Model” (54, 55); with permission from Wirnitzer et al. (1).

The dual HEAL approach is a scientifically approved strategy due to its well-known, unique, and promising health benefits (28–31, 42, 43). This approach emphasizes the codependent and continuous application of healthy dietary patterns (1, 44, 45) and regular engagement in PA and exercise (1, 45–47), which primarily serves to address the dynamic interplay between energy intake and expenditure (48, 49). This dual approach highlights the significance of a holistic perspective that integrates various dimensions and interactions to generate network models of health and disease functioning (30, 42). Therefore, HEAL displays the minimum recommendation to unlock and begin tapping into the full potential of the power of lifestyle to improve one’s health at the individual level from childhood (even from prenatal life) to adulthood and old age (50). HEAL can contribute to prioritizing health and wellbeing over disease (i.e., person and/or patient-centered over disease and/or medication-centered approach) with the projected potential to shift to a preventive person-centered health approach. Derived from models of the determinants of health (51–55), the most relevant target groups considering personal and public health to be addressed span all levels and units of action to achieve better public health in nations that emerge from improved individual health, i.e., from the micro-level over the meso-level, to the macro-level (Figure 2).

While the power of HEAL holds great promise for improving health and wellbeing, access to healthy food and opportunities for regular PA are often influenced by various broader environmental and societal factors (56, 57). For example, key barriers such as concerns about food safety, air quality, and the environment limit people’s ability to meet dietary and PA guidelines, particularly in under-resourced or densely urbanized areas (57). In addition, affordability remains a significant challenge, as healthy, fresh foods and safe opportunities for regular PA are often less available or more expensive in low-income communities, further exacerbating health inequalities (58, 59). Cultural preferences and traditions also influence food choices and PA behaviors, highlighting the need for public health strategies that (i) are culturally sensitive and inclusive (60, 61) and (ii) should aim to respect local dietary patterns and promote culturally relevant forms of PA, thereby encouraging better adherence to health recommendations.

Childhood, adolescence, and emerging adulthood are susceptible life stages for ingraining the core concepts of healthy behavior. However, the challenge of the behavior change process remains significant for adults to cope with, and considering that relapses may occur (and at various stages), the successful adoption of new healthy behavior (Stages 1–5: precontemplation, contemplation, preparation, action, maintenance) and the complete termination of previous unhealthy behavior is rare (Stage 6) (62, 63). Therefore, defining behavior change success is suggested as any forward progression in the model, rather than focusing solely on reaching the final stage of termination. Indeed, when the power of personal behavior at its lowest range estimate (the cause of 40% of all deaths) exceeds all other determinants of health, including the healthcare system (10%), environment (5%), social circumstances (15%), and genetics (30%), the pursuit of tackling this challenge becomes even more crucial (51–55, 64).

**Figure 3:**
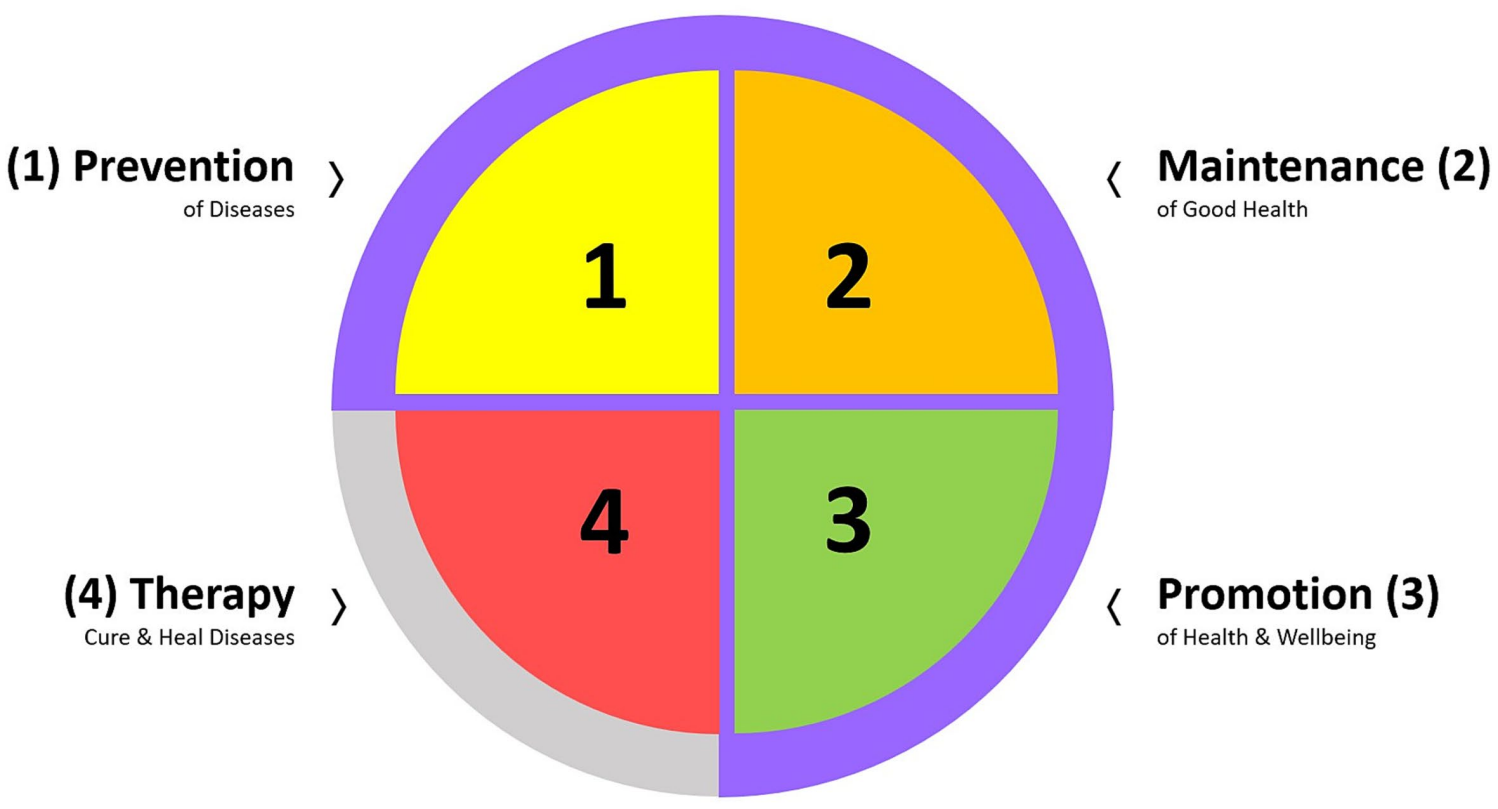
The four areas of action comprise health-oriented action, competence, readiness to achieve lifelong health and wellbeing, and sustainable care. © Katharina C. Wirnitzer.

Preventive and therapeutic models can surpass empirical reductionism by embracing lifestyle-oriented preventive strategies (i.e., HEAL) (65, 66). To meet this, it seems crucial for any therapeutic approach to consider and effectively apply the 3:1 ratio (Figure 3): three competence-oriented areas of health-related actions aimed at empowering individuals, including—first and foremost—the prevention of diseases, maintenance of good health status, and promotion of health, followed by one, as a last resort, medicalized therapeutic strategies to target specific health conditions, including curing and healing diseases. Consistent with this “Prevention First” appeal (acknowledged across many global health policies but with deficient implementation) (67–71), EU policymakers have already identified and emphasized (i) the urgent need for greater and more pressing efforts toward a shift to the prevention of ill-health and disease with health promotion as a key pillar in ensuring future public health (67–70), and (ii) the cross-European need to focus on changes in future medical and health pedagogy to address the current public health gaps (71, 72).

Now that the world has passed the COVID-19 pandemic, there is no guarantee that hidden health-related concerns or consequences (especially those associated with psychosocial health) will not emerge or develop, or that the world will not experience a similar health tragedy. While current global policies face challenges in effectively proposing and addressing Earth’s overpopulation as a general approach to mitigate most global pandemics, there appears to be a further emphasis on the fundamental adoption of healthy lifestyle behaviors as a critical factor in modern and sustainable health and care practices for better public health outcomes to address existing public health concerns. Nevertheless, the COVID-19 pandemic has revealed that the individual’s health is not equal to the community’s health. Therefore, at the very least, benefits from behavioral change occur across societal levels. From the perspective of the unenthusiastic population health developments worldwide, the public health community urgently needs the willingness to act from professionals of all health-related areas, spheres, and levels of influence with advice-focused, hands-on techniques, along with the drivers of those generating increasing social, health, and care inequalities for fairer treatment in the post-pandemic world (39).

Given the importance of informed lifestyle choices as a practical step (67–72), health experts, policymakers, journalists, and patients should be conscious of corporate-sponsored material regarding the nature or prevalence of the disease. Instead, they should rely on evidence-based, publicly funded sources that provide information on lifestyle-oriented strategies for the prevention and treatment of common health problems as a primary preventive course of action. Likewise, rather than relying solely on prescribed medication, it is essential to consider the power of lifestyle medicine and health behavior, prioritizing a person-centered health approach over a disease-centered one (42). This involves making healthy lifestyle choices the easy choices with the first line of intervention, prioritized above the immediate prescription of medications to address unhealthy behaviors (28, 29). Collectively, it is also essential for politicians, physicians, the media, and the public to address the disparities and conflicts between public and private health, including issues such as the right to health and patient autonomy. Rather than debating slogans, it is key to identify tailored solutions to the pressing health issues and challenges.

To implement these priorities in line with Figures 1–3, structural actions are needed across medical, educational, and policy systems. Therefore, Table 2 provides a brief policy roadmap (including 10 milestones) for key protagonists to address today’s health paradox and start unfolding the power of lifestyle (39, 73) arising from HEAL. The steps and strategies suggested are based on sound evidence from public health, resonating with the ethical responsibility of healthcare systems to prioritize prevention over treatment of chronic diseases to proliferate joint actions of stakeholders (i.e., policy and decision makers), communities, and individuals in tackling the issues of most pressing concern today. Thus, it is envisioned that a coordinated framework enabling Health in All Policies (HiAP) (74) is essential for nationwide hands-on measures to connect individual actions with broader systemic change. The roadmap delivers successful horizontal (professional/specialized disciplines and occupational peer groups) and vertical permeability (individual/patient, mass-media) of evidence-based knowledge and competencies (75, 76) at all levels (macro ↔ meso ↔ micro) and public settings for maximal effectiveness regarding health promotion and the prevention of ill-health and NCDs. Nevertheless, the consensus process also highlighted that a substantial proportion of the 33 statements had an evidence score corresponding to the lowest tier within the categories rating the strength of evidence. This apparent paradox (strong conceptual agreement despite limited high-level empirical evidence) helps explain why progress in implementing effective prevention strategies remains slow. Several systemic factors contribute to this discrepancy, including (i) incomplete evidence bases for complex, multilevel lifestyle interventions; (ii) a persistent lack of confidence among policymakers that long-term preventive benefits will outweigh the short-term financial and structural costs; and (iii) increasing societal demand for health and social services occurring in parallel with already limited public-health resources. These factors collectively offer a partial explanation for why advances in health science and rising healthcare expenditures have not yet translated into proportionate improvements in the public health of nations, with the latter posing a central question underpinning this roadmap. Addressing today’s health paradox, therefore, requires not only generating high-quality evidence but also overcoming structural, economic, and political barriers that impede the adoption of evidence-based lifestyle-centered prevention strategies.

At the policy level, measures such as regulating marketing practices, improving public catering and school/university meal standards, and supporting community-based health and wellbeing initiatives can create supportive conditions for lasting behavioral change. The fact that ‘Health Promotion’ is an overarching learning objective and teaching principle and is relevant to all educational levels and health-related disciplines requires school and tertiary curricula to be upgraded in order to empower future generations with conscious, holistic, and sustainable health behavior at a modern rate, and to qualify future doctors and teachers on the basics of the power of lifestyle (HEAL). Integrating lifestyle-centered modules seamlessly into the training of healthcare professionals (doctors, nurses) and educators (teachers/pedagogues, professors) for up-to-date counseling and treatment considering informed lifestyle decisions (39, 73, 75–77) is therefore crucial. Continuous health development of professionals (e.g., advanced training) is essential to ensure that health experts, healthcare providers, and educators stay updated with emerging evidence and can confidently recommend non-pharmacological lifestyle-oriented interventions as the first line of treatment. At the community level, infrastructure changes that facilitate access to healthy and affordable food, active transport, opportunities that motivate and stimulate regular PA and exercise engagement, healthy public spaces, and support of community-based health and wellbeing initiatives are key to reducing the environmental and social barriers that often undermine healthy behaviors (78). Tailored community-level interventions should be aligned with national health promotion strategies, such as culturally adapted HEAL programs. Clinical routines should include regular assessment of lifestyle risk factors using validated tools, alongside personalized counseling, and a comprehensive monitoring and feedback system can help evaluate progress and guide future interventions. Together, these measures can support a shift toward a more prevention-centered medical culture that puts individual empowerment and the resulting population-level health outcomes at its core.

Interested readers may also consult the comprehensive consensus report (1) for more detailed information.

## 5 Conclusion

This short policy report summarizes the conclusions of a structured ACCORD-based consensus-statement process, informed by three events held as international, multidisciplinary meetings in Innsbruck, Austria (2020–2022), which was further developed by 58 internationally recognized experts who were senior specialists in their respective fields and engaged primarily through structured remote exchanges throughout the entire process. They identified gaps in current health-related approaches, strategies, and research priorities and approved 33 evidence-based consensus statements along with a policy roadmap to address today’s health paradox.

In conclusion, it is the consensus of the panel of experts that the power of lifestyle medicine can significantly contribute to the “Prevention First” appeal, and that the dual HEAL approach to sustainable and lifelong health is the minimum recommendation to promote individual and public health. The expert panel also proposes that future health policies and studies prioritize integrated lifestyle-centered strategies, stronger health literacy, and improved methodological standards to support sustainable, lifelong health across diverse populations.

## Author disclaimer

The views, opinions, and findings expressed within this multidisciplinary consensus statement study and contained in this report are those of the authors and should not be construed or interpreted as representing the official viewpoint, guideline or decision of the Department of Research and Development in Teacher Education of the University College of Teacher Education Tyrol, Innsbruck, Austria, or representing exhaustingly the state-of-the-art science from the cross-cut area at the interface of or overlapping/inter-disciplinary areas medicine and health sciences, life sciences, sports science, and nutrition science, respectively. Approved for public release; distribution unlimited.
